# Midline 1 controls polarization and migration of murine cytotoxic T cells

**DOI:** 10.1002/iid3.44

**Published:** 2015-01-27

**Authors:** Lasse Boding, Ann K Hansen, Morten M Nielsen, Germana Meroni, Thomas H Braunstein, Anders Woetmann, Niels Ødum, Charlotte M Bonefeld, Carsten Geisler

**Affiliations:** 1 Department of International Health, Immunology and Microbiology, Faculty of Health and Medical Sciences, University of CopenhagenCopenhagen, Denmark; 2 Institute for Maternal and Child Health-IRCCS “Burlo Garofolo”Trieste, Italy; 3 Danish National Research Foundation Centre for Cardiac Arrhythmia, Department of Biomedical Sciences, Faculty of Health and Medical Sciences, University of CopenhagenCopenhagen, Denmark

**Keywords:** contact hypersensitivity, CTLs, microtubule, Midline 1, migration

## Abstract

Midline 1 (MID1) is a microtubule-associated ubiquitin ligase that regulates protein phosphatase 2 A levels. Loss-of-function mutations in MID1 lead to the human X-linked Opitz G/BBB (OS) syndrome characterized by defective midline development during embryogenesis. We have recently shown that MID1 is strongly up-regulated in murine cytotoxic T lymphocytes (CTLs), and that it has a significant impact on exocytosis of lytic granules and the killing capacity of CTLs. The aims of the present study were to determine the localization of MID1 in migrating CTLs, and to investigate whether MID1 affects CTL polarization and migration. We found that MID1 mainly localizes to the uropod of migrating CTLs and that it has a substantial impact on CTL polarization and migration in vitro. Furthermore, analysis of contact hypersensitivity responses supported that MID1 controls effector functions of CTLs in hapten-challenged skin in vivo. These results provide significant new knowledge on the role of MID1 in CTL biology.

## Introduction

Cytotoxic T lymphocytes (CTLs) play a key role in the defense against virus infections. To recognize infected cells in peripheral tissues and clear infections, circulating CTLs extravasate and migrate to infected sites in a highly regulated manner. CTLs are sensitive to special adhesion molecules and chemoattractants produced in the inflamed tissue and migrate by inducing rearrangement of the actin and microtubule cytoskeleton 1–5. Rearrangement of the cytoskeleton in migrating T cells causes elongation and polarization of the cells in the directional axis of motility with a leading edge in the front and a uropod in the rear 1–5. Directional cell migration requires protrusion of the leading edge and retraction of the uropod, processes that are driven by actin polymerization and actomyosin contraction and that are not directly dependent on microtubules 3,6–8. However, microtubule dynamics affect actin polymerization and thereby cell migration 4,6,7,9–11. In addition to cell migration, microtubules play important roles in cell polarization, vesicular transport and in cell division, and it has been demonstrated that agents that depolymerize microtubules inhibit T cell polarization, migration, and cytotoxicity 4,5,12. Microtubules are hollow tubes made up by protofilaments composed of α- and β-tubulin dimers. The two ends of a microtubule are not equal. The end where α-tubulin is exposed is called the minus end, and it is usually embedded in the microtubule-organizing centre (MTOC) also called the centrosome, whereas the end where β-tubulin is exposed is called the plus end. The plus end alternates between two phases of growth and shrinkage and is therefore also called the dynamic end 6,7. The dynamics of microtubule plus ends are extensively regulated, and many regulatory factors that promote microtubule growth or shrinkage have been described 13.

Midline 1 (MID1) is an ubiquitin ligase associated with the microtubules 14–16 that regulates the level of microtubule-associated phosphatase 2 A (PP2A) 17,18. Mutations in the RING finger domain of MID1 cause the X-linked form of the Opitz G/BBB syndrome (OS) that is characterized by disorders that primarily affect the ventral midline 19. An accumulation of the catalytic subunit of PP2A (PP2Ac) is found in fibroblasts from patients with OS due to impairment of the ubiquitin ligase activity of the mutated MID1. The increased level of PP2Ac is associated with reduced phosphorylation of microtubule-associated proteins that regulate microtubule dynamics 17. From these observations it has been suggested that MID1 plays crucial roles during embryogenesis by regulating microtubule dynamics 20,21.

As MID1 controls MTOC polarization, exocytosis of lytic granules and killing of target cells 22, we speculated that MID1 affects microtubule dynamics in CTLs and thereby CTL migration. The aims of the present study were to determine the localization of MID1 in migrating CTLs and to examine whether MID1 affects CTL polarization and migration. To this end, we studied CTLs from P14 TCR transgenic mice and P14 mice crossed to MID1^-/-^ mice (P14MID1^-/-^ mice). We show that MID1 primarily localizes to the uropod of migrating CTLs and has a substantial impact on CTL polarization and migration in vitro and in contact hypersensitivity responses in vivo.

## Materials and Methods

### Mice

Generation of the MID1^-/-^ mouse strain has been described 22,23. The P14 mice (transgenic line 318) express the transgenic Vα2Vβ8 TCR specific for the lymphocytic choriomeningitis virus glycoprotein gp_33–41_ bound to H-2D^b^ 24,25. The MID1^-/-^ and P14 strains were on a C57BL/6 background. MID1^-/-^ mice were crossed to P14 mice to generate P14MID1^-/-^ mice. The animal experiments were approved by the Animal Experiments Inspectorate, The Danish Ministry of Justice (approval number 2007/561-1357 and 2012-15-2934-00663).

### Generation of CTLs and FACS analysis

P14 and P14MID1^-/-^ CTLs were generated by stimulating splenocytes isolated from TCR transgenic P14 and P14MID1^-/-^ mice with 5 ng/mL of lymphocytic choriomeningitis virus glycoprotein gp_33–41_ (Schafer-N, Copenhagen, Denmark) for three days in complete medium (RPMI 1640 with 10% FBS, 1% l-glutamine, 0.5 IU/l penicillin, 500 mg/L streptomycin, 50 µM 2-mercaptoethanol) supplemented with 20 U/mL IL-2 and subsequently resting them for two days as previously described 26. For FACS analyses we used antibodies against CD8 (53–6.7), TCRVα2 (B20.1), CD62L (MEL-14), CD44 (IM7), LFA-1 (2D7), CCR5 (C34–3448), and CCR7 (4B12) all from BD Biosciences, San Jose, CA, USA. Anti-CXCR6 (221002) was from R&D Systems, Minneapolis, MN, USA, and anti-CXCR3 (173) and anti-CCR6 (29–2L17) from Biolegend, San Diego, CA, USA. For weakly expressed markers, cells were incubated up to one hour at 37 °C. Otherwise all staining was done at 4 °C for 20 min. Acquisition was done on a LSRFortessa flow cytometer from BD Biosciences. Data were analyzed using FlowJo (Treestar, Ashland, OR, USA) software.

### MID1 localization, CTL morphology and migration, and ezrin staining

To determine MID1 localization in live migrating CTLs, 5 × 10^6^ P14MID1^-/-^ CTLs were transfected with 4 μg of a plasmid encoding a chimeric MID1-GFP molecule (a gift from Susann Schweiger 15) as previously described 27,28. Migration of the MID1-GFP transfected cells over poly-l-lysine coated coverslips was recorded in time series experiments with images taken every 5 sec using a Zeiss LSM 780 confocal microscope with a 63x/1.4 NA oil objective and a XL S1 incubator.

CTL morphology and migration were visualized by DIC images on live CTLs plated on poly-l-lysine coated coverslips. For morphology, the DIC images were blinded and the cells stitched using a pen tablet (Wacom, Saitama, Japan) and Fiji imaging software (www.fiji.sc). Circularity and perimeter were calculated by the Measure command in Fiji, where the perimeter equals the length of the outside boundary of the selected cell, and the circularity is calculated as (4π × area/perimeter^2^). For migration experiments, CTL movement was recorded in time lapse experiments. Samples were blinded and motility of the cells was calculated after tracking their path using Fiji software and the Manual Tracking command.

For ezrin staining, CTLs were plated on poly-l-lysine coated coverslips for 30 min at 37 °C, fixed in 2% paraformaldehyde, permeabilized in 0.2% Triton-X, and incubated with rabbit anti-ezrin antibody (clone 3145, Cell Signaling, Danvers, MA, USA) for 45 min. The cells were subsequently incubated with Alexa Fluor 488-conjugated goat anti-rabbit Ig antibody (A11034, Invitrogen, Eugene, OR, USA) for 30 min, mounted and imaged. All procedures were performed at room temperature.

### Transwell migration

For transwell experiments, CTLs were pre-incubated in RPMI with 0.5% bovine serum albumin for 90 min, placed in the upper chamber of a 96-well transwell plate with a pore size of 5 μm (Corning, Tewksbury, MA, USA) and allowed to migrate towards the bottom chamber containing the indicated concentrations of FBS for three hours. FBS was used as a general chemoattractant as suggested by the transwell system manufacturer (Corning). The fraction of cells that had migrated to the bottom chamber was subsequently determined by FACS analysis. The specific migration is given as the fraction of migrated cells towards the indicated concentration of FBS subtracted the fraction of migrated cells towards medium without FBS.

### Hapten sensitization and elicitation of contact hypersensitivity responses

C57BL/6 and MID1^-/-^ mice were sensitized with 25 µL 0.15% DNFB in a vehicle composed of a 1:4 olive oil:acetone mixture on the dorsal side of both ears for three consecutive days (0–2) as previously described 29,30. On day 23, the mice were challenged on the dorsal side of both ears with 25 µL 0.15% DNFB in vehicle. Control C57BL/6 and MID1^-/-^ mice were sensitized on day 0–2 and challenged on day 23 with the pure vehicle. Twenty-four hours prior to challenge mice were given 0.8 mg/mL bromodeoxyuridine (BrdU, BD Biosciences) in their drinking water. Mice were euthanized 24 h after challenge and the ear inflammation was assessed by measuring the ear thickness using an engineer's micrometer (Mitutoyo, Tokyo, Japan). Relative ear thickness is given as the ear thickness of DNFB challenged ears relative to the ear thickness of vehicle challenged ears. The draining lymph nodes (LN) were harvested, and single-cell suspensions were prepared for FACS analysis and stained for CD8 expression and BrdU incorporation as previously described 31. The left ears were individually homogenized in a Precellys 24 tissue homogenizer (Bertin Technologies, Montigny-le-Bretonneux, France), and total RNA was extracted with a RNeasy kit (Qiagen, Hilden,Germany) as previously described 32. The samples were DNase treated and RNA concentrations were measured using a Nanodrop 2000 C Spectrophotometer (Thermo Scientific, Roskilde, Denmark). Samples were adjusted to 2000 ng/μL, converted to cDNA (High Capacity RNA-to-cDNA kit, Applied Biosystems, Grand Island, NY, USA) and used as template in TaqMan QPCR testing for CD8a (Mm01182107_g1, #4331182) and GAPDH (Pre-Developed TaqMan Assay Reagents, Mouse GAPDH (20x) #4352932E) (Life Technologies, Grand Island, NY, USA) expression using a Stratagene Mx3005P (Agilent Technologies, Santa Clara, CA, USA). The right ears were kept on ice until embedded in Tissue-Tek (Sakura, Torrance, CA, USA) and snap frozen in isopentane cooled with dry ice. The ears were cut in 20 µm transverse sections using a Microm HM525 Cryostat (Termo Scientific, Grand Island, NY, USA) at −22 °C, stained over-night with anti-mouse CD8a antibodies (clone 53–6.7, BD Biosciences), incubated with secondary goat anti-rat IgG Alexa Fluor 488 (Life Technologies) and Hoechst 33342 (Invitrogen), and mounted with Dako Fluorescence Mounting Medium (Dako, Glostrup, Denmark). Whole ear imaging was performed on a Zeiss AxioScan.Z1 Slice Scanner using a 20x/0.8 plan apochromat objective. The whole depth of the specimens was scanned by doing a z-stack in each x-y position. Afterwards, a maximum intensity projection was performed to visualize the strongest signals from all planes. Tile scans were set up to cover the whole ear, and stitching was performed to fine tune alignment. High quality images of inflammatory areas were taken on a Zeiss LSM780 confocal microscope using a 20×/0.8 objective. All data analysis and presentation was performed in Zen Blue or Black (Carl Zeiss, Jena, Germany).

### Statistical analysis

Statistical analyses were performed using Student's *t*-test or Mann–Whitney test with a 5% significance level, unpaired observations and equal variance.

## Results

### MID1 primarily localizes to the uropod of migrating CTLs

**Figure 1:**
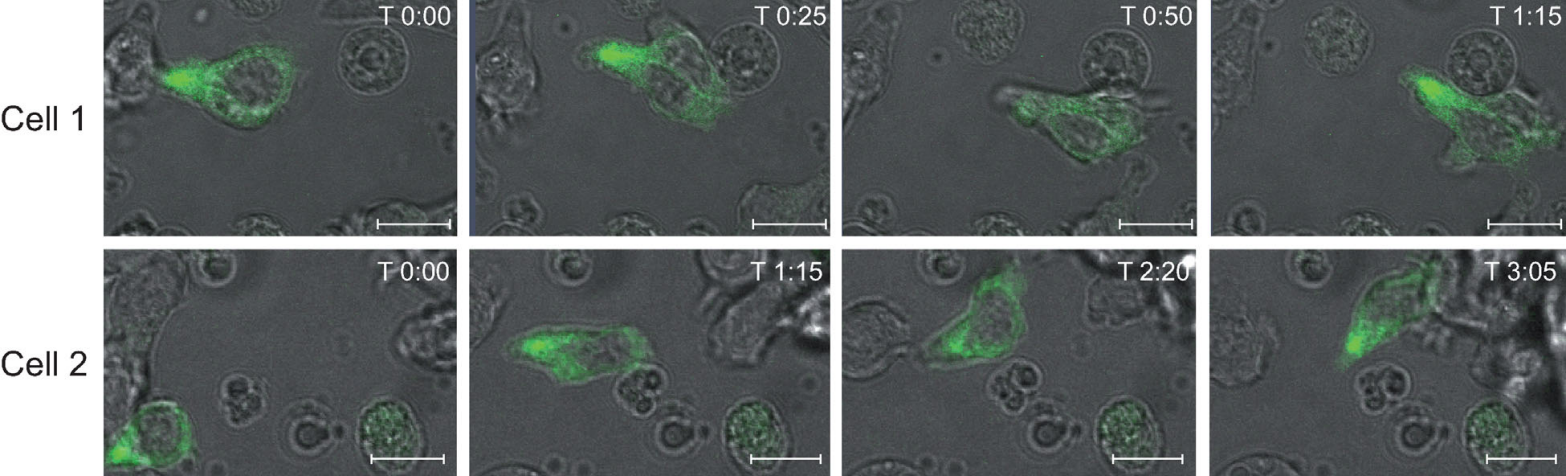
MID1 primarily localizes to the uropod in migrating CTLs. Localization of MID1 in two migrating P14MID1^-/-^ CTLs transfected with MID1-GFP and followed by live imaging using a Zeiss LSM780 confocal microscope with a 63× objective and a XL S1 incubator. Time (T) in minutes:seconds is shown at the top right of each panel. The scale bars are 10 μm. Two representative cells of four independent experiments are shown. Supporting Information Video 1 and 2 show the complete time lapse series of these two cells.

In most types of migrating cells, the MTOC is localized in front of the nucleus with the majority of microtubules radiating towards the leading edge 6–8. In contrast, in migrating T cells the MTOC is located behind the nucleus in the uropod together with the majority of the microtubules and only a minor fraction of the microtubules radiates out from the MTOC in the uropod to the leading edge 1–3,5. This special localization of the MTOC and microtubules in T cells has been proposed to facilitate the deformability required for their migration through constricted spaces 1–3. Previous studies have found that MID1 is associated to the microtubules in human and mouse fibroblasts and in COS-7, MCF-7, and HeLa cells transfected with MID1-GFP 14–16. To determine the localization of MID1 in migrating CTLs, we transfected P14MID1^-/-^ CTLs with plasmids encoding chimeric MID1-GFP molecules and followed the migratory pattern of the transfectants by use of live confocal imaging. We found that MID1 primarily localizes to the uropod of migrating CTLs (Fig. 1 and Supporting Information Video 1 and 2). This observation supported that MID1 is associated with the microtubules in CTLs as found for other cell types, and that MID1 might be involved in cell migration.

### MID1 controls CTL polarization

**Figure 2:**
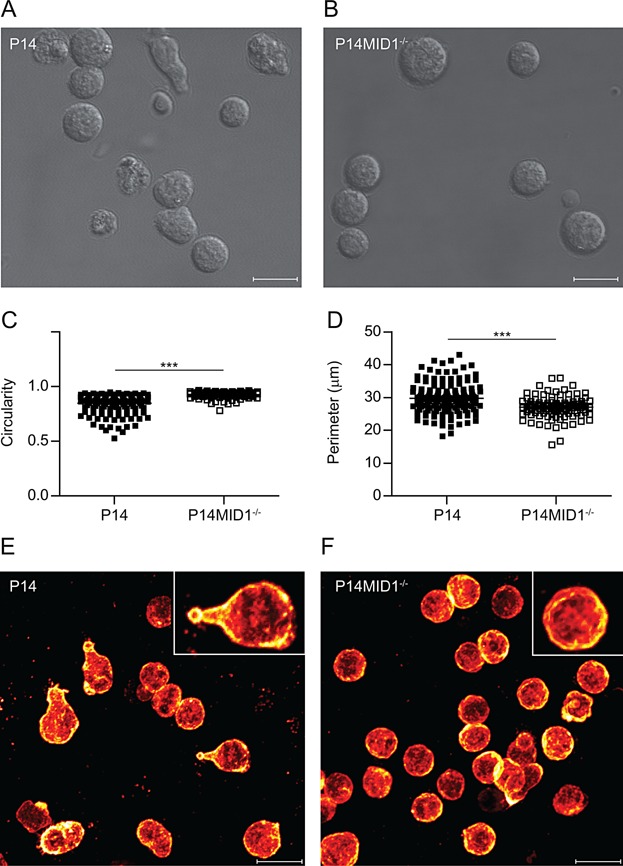
MID1 controls CTL morphology. (A) Representative DIC images from confocal microscopy of P14 and (B) P14MID1^-/-^ CTLs from four independent experiments. (C) Circularity and (D) perimeter analyses of P14 (*n* = 161) and P14MID1^-/-^ (*n* = 116) CTLs pooled from four independent experiments. (E) Representative images of ezrin expression in P14 and (F) P14MID1^-/-^ CTLs from four independent confocal microscopy experiments. ^***^denotes Student's *t*-test with *P* < 0.0001. The scale bars are 10 μm.

Directional T cell migration requires polarization and elongation of the cell with a leading edge in the front and a uropod in the rear 1–5. To explore the influence of MID1 on CTL polarization, we plated live P14 and P14MID1^-/-^ CTLs on poly-l-lysine coated coverslips and visualized their morphology by differential interference contrast (DIC) images using a confocal microscope. We found that P14 CTLs varied considerably in morphology from ruffled spherical to elongated, whereas P14MID1^-/-^ CTLs appeared more homogeneous with a smooth, spherical shape (Fig. 2A and B). To quantify differences in the morphology between P14 and P14MID1^-/-^ CTLs, we measured the circularity and perimeter of the cells in blinded samples. These analyses confirmed that P14MID1^-/-^ CTLs had a higher degree of circularity (Fig. 2C) and were less ruffled and elongated as determined by a smaller perimeter (Fig. 2D).

Specific proteins, including the ezrin/radixin/moesin (ERM) proteins, have been shown to segregate to the T cell uropod 3,33,34. To further study the role of MID1 in CTL polarization we plated P14 and P14MID1^-/-^ CTLs on poly-l-lysine coated coverslips and subsequently stained them with antibodies against ezrin. We found that ezrin segregated to uropod-like structures in several P14 CTLs, whereas it was evenly distributed in P14MID1^-/-^ CTLs that did not form uropods (Fig. 2E and F). Taken together these observations indicated that MID1 controls CTL polarization and uropod formation.

### MID1 controls CTL migration

**Figure 3:**
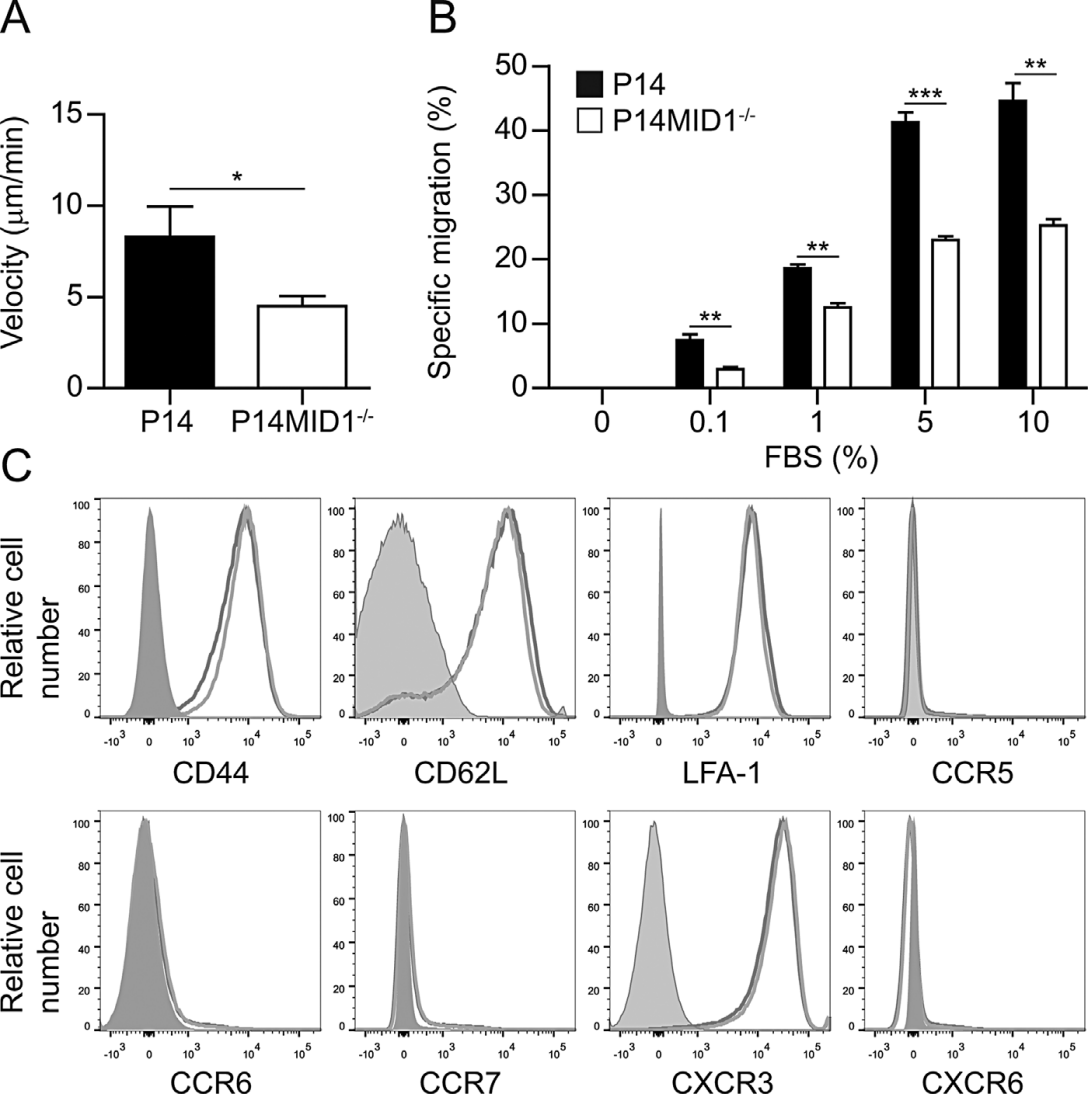
MID1 controls CTL migration. (A) The velocity of P14 (*n* = 40) and P14MID1^-/-^ (*n* = 73) CTLs migrating over poly-l-lysine coated coverslips. Confocal microscopy time lapse series were blinded and the migratory paths analyzed with Fiji software. Bars show mean values ± SEM from four independent experiments. (B) Specific migration of P14 and P14MID1^-/-^ CTLs through transwell filters towards the indicated FBS concentration. Shown are mean values ± SEM from one representative experiment out of three. ^*^ and ^***^ denotes Student's *t*-test with *P* < 0.05 and *P* < 0.0001, respectively. (C) FACS histograms of P14 (blue) and P14MID1^-/-^ (red) CTLs stained with antibodies against the indicated adhesion molecules and chemokine receptors. One representative histogram from at least three independent experiments is shown for each staining. The gray curves show fluorescence minus one (FMO).

To study whether MID1 affects CTL migration, we first measured the speed of migration of P14 and P14MID1^-/-^ CTLs over poly-l-lysine coated coverslips. We found that P14MID1^-/-^ CTLs had a reduced migration speed compared to P14 CTLs (Fig. 3A). To further study the effect of MID1 on CTL migration, we plated P14 and P14MID1^-/-^ CTLs in the upper chamber of transwell chambers with a pore size of 5 µm and allowed them to migrate towards a fetal bovine serum (FBS) gradient for 3 h. We found that a significant smaller fraction of P14MID1^-/-^ CTLs migrated through the filters compared to P14 CTLs (Fig. 3B). To exclude that the migratory defect of P14MID1^-/-^ CTLs was simply due to an altered expression of adhesion molecules and/or chemokine receptors, we did a FACS analyzed of the cells with a panel of antibodies against these markers; however, we did not find any differences in the expression of adhesion molecules or chemokine receptors between P14 and P14MID1^-/-^ CTLs (Fig. 3C). Taken together, these results indicated that MID1 affects CTL migration.

### MID1 regulates contact hypersensitivity responses

**Figure 4:**
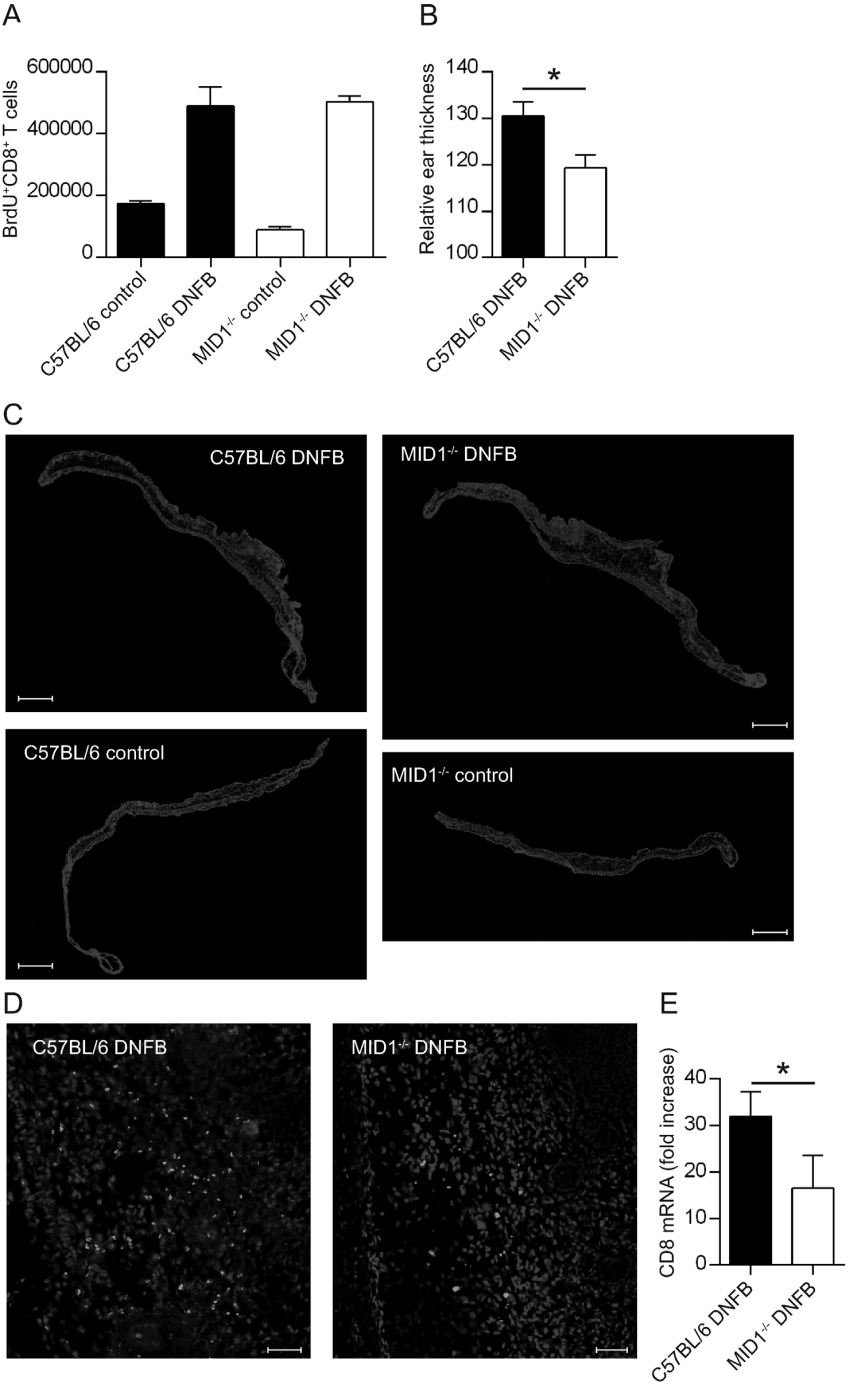
MID1 regulates contact hypersensitivity responses. (A) Numbers of BrdU^+^CD8^+^ T cells in the draining LN of C57BL/6 and MID1^-/-^ mice challenged with vehicle (control) or DNFB measured by FACS analysis. Shown are mean values ± SEM from one representative experiment out of two with four mice in each group. (B) Relative ear thickness in C57BL/6 and MID1^-/-^ mice challenged with DNFB. Shown are mean values ± SEM from two independent experiments with a total of 16 ears in each group. ^*^Denotes Student's *t*-test with *P* < 0.05. (C) Whole-ear immunofluorescence scans. The nuclei stained with Hoechst are shown in blue and CD8 staining is shown in green. The top row shows ears from mice treated with DNFB and the bottom row ears from mice treated with vehicle (control mice). Scale bars are 1 mm. Please see Supporting Information Figure 1–4 for high resolution versions. (D) High quality confocal images of inflammatory areas in the ears of mice treated with DNFB. Scale bars are 50 µm. (C, D) Images are representative of at least two independent experiments. (E) QPCR analysis showing fold increase in CD8 mRNA expression in whole ears of DNFB-treated mice relative to CD8 mRNA expression in whole ears of vehicle-treated mice. ^*^Denotes Mann–Whitney test with *P* < 0.05. Data show mean values ± SEM from two independent experiments with a total of 7 mice in each group.

Contact hypersensitivity is a T cell-mediated response to allergens/haptens. Specific T cells are activated during sensitization with haptens such as 2,4-dinitrofluorobenzene (DNFB) and CTLs are the primary effector cells mediating the contact hypersensitivity response following re-exposure to the hapten 35–39. Thus, exposure to DNFB in individuals already sensitized to DNFB induces migration of hapten-specific CTLs into the skin challenged with DNFB. In the challenged skin the CTLs are activated and induce the inflammatory response. In a widely used animal model for contact hypersensitivity, mice are sensitized and challenged on the ears with haptens/allergens such as DNFB and the ensuing inflammatory response is then measured by determining the swelling of the ears 29–31,40,41. To investigate the role of MID1 in CTL responses in vivo, we sensitized C57BL/6 and MID1^-/-^ mice with DNFB on their ears for three consecutive days and challenged them with DNFB on their ears three weeks later. Control C57BL/6 and MID1^-/-^ mice were sensitized and challenged with the pure vehicle. Twenty-four hours after challenge, the mice were killed and the numbers of proliferating CD8^+^ T cells in the draining LN and the ear thickness were determined as previously described 29–31. We found that challenge with DNFB increased the numbers of proliferating CTLs as defined as BrdU^+^CD8^+^ cells to the same extend in the draining LN of C57BL/6 and MID1^-/-^ mice (Fig. 4A). Thus, MID1 did not seem to affect primary activation of CD8^+^ T cells in the LN. In contrast, when we measured ear thickness, we found that the ears of MID1^-/-^ mice challenged with DNFB were markedly less swollen than the ears of C57BL/6 mice challenged with DNFB (Fig. 4B), indicating that MID1 affects CTL responses in peripheral tissues. To investigate whether the reduced swelling of the ears of MID1^-/-^ mice treated with DNFB could be caused by an impaired migration of CTLs to the ears, we next attempted to enumerate CD8^+^ T cells in DNFB-treated and control ears from C57BL/6 and MID1^-/-^ mice by immunofluorescence staining. To visualize transverse sections of whole ears we did tile scans using a high-throughput slide scanner (Fig. 4C and Supporting Information Fig. 1–4). We observed a high degree of CD8^+^ T cell infiltration in DNFB-treated ears from both C57BL/6 and MID1^-/-^ mice compared to the ears from vehicle-treated control mice. Analyses using higher magnification indicated a reduced infiltration of CD8^+^ T cells in ears of MID1^-/-^ mice compared to C57BL/6 mice (Fig. 4D). To more precisely quantify the CD8^+^ T cell infiltration, we subsequently measured the mRNA expressions levels of the CD8a gene in the ears by QPCR. We calculated the fold increase of CD8a mRNA in DNFB-treated mice compared to control mice, and found a significantly lower CD8a mRNA up-regulation in MID1^-/-^ ears compared to C57BL/6 ears (Fig. 4E). Taken together, these experiments indicated that MID1 controls migration of CD8^+^ T cells during inflammatory responses in vivo.

## Discussion

In this study we show that MID1 controls polarization and migration of CTLs. MID1 is a microtubule-associated ubiquitin ligase that regulates the level of microtubule-associated PP2A in embryonic fibroblasts 14,15,17,18. MID1 is causally linked to OS and plays important roles in the formation of ventral midline structures during embryogenesis 19. Whether MID1 plays any role after embryogenesis was unknown until recently, where it was found that MID1 is involved in induction of allergic airway inflammation in the bronchial epithelium 42. Furthermore, we recently found that MID1 is up-regulated in CTLs where it directs lytic granule exocytosis and cytotoxicity 28. In the present study we found that MID1 predominantly localizes to the uropod and that a minor part radiates out from the uropod towards the leading edge in migrating CTLs. This localization of MID1 overlaps with the previously described localization of microtubules in migrating T cells 1–5. Taken together with the observations that MID1 is associated with the microtubules in fibroblast and in various cell lines transfected with MID1 14–16, our results indicate that MID1 also associates with the microtubules in CTLs.

Previous studies have described that microtubule dynamics regulate the morphological polarization and migration of T cells. Thus, T cells treated with nocodazole that disrupts microtubules are unable to form a stable uropod and a leading edge and exhibit impaired migration 4, and likewise T cells with knocked-out mammalian diaphanous-related formin that stabilizes the microtubules are also unable to morphologically polarize and show impaired migration 5. We found that acquisition of a polarized morphology is profoundly impaired in CTLs from P14MID1^-/-^ mice and that MID1 is required for normal CTL migration both over coverslips and through transwell filters. Combined with the studies mentioned above 4,5 our data indicate that MID1 controls CTL polarization and migration by affecting microtubule dynamics. This is further supported by the observation that MID1 regulates PP2A and phosphorylation of microtubule-associated proteins in fibroblasts 17. However, further studies are required to directly determine if and how MID1 affects microtubule dynamics.

Recently, it was shown that microtubule dynamics are involved in the in vivo migration of specific T cells to allergen-challenged skin 5. To study whether the impaired polarization and migration of P14MID1^-/-^ CTLs in vitro translated into altered CTL responses in vivo, we studied the inflammatory response in skin challenged with the hapten DNFB. This is a well-characterized model 29,30, where hapten-specific CTLs are the primary effector cells mediating the inflammatory response following re-exposure of the skin to DNFB 35–39. We found that the inflammatory response elicited by DNFB, as measured by ear swelling and CD8^+^ T cell infiltration, was significantly impaired in MID1^-/-^ mice compared to C57BL/6 mice. In contrast, the primary response in the draining LN was not affected in MID1^-/-^ mice. At first sight this may seem to be a paradox; however, we have recently shown that MID1 is weakly expressed in thymocytes and naïve T cells, and that it does not affect activation of naïve CD8^+^ T cells in vitro 28. Thus, our data supported that the impaired polarization and migration of MID1^-/-^ CTLs translated into altered CTL responses in vivo and confirmed that MID1 does not affect activation of naïve CD8^+^ T cells.

In conclusion, our data demonstrate that MID1 contributes to the regulation of CTL polarization and migration. While the molecular pathways whereby MID1 impacts on CTL polarization and migration require further investigations, our data suggest that MID1 plays a significant role in migration-dependent CTL responses most probably by affecting microtubule dynamics. Thus, MID1 directs multiple events in CTL responses adding a new pathway to the regulation of CTLs.

## Supporting Information

**Video 1 & 2** MID1 primarily localizes to the uropod in migrating CTLs. Localization of MID1 in migrating P14MID1 CTLs transfected with MID1-GFP and followed by live imaging using a Zeiss LSM780 confocal microscope with a 63x objective and a XL S1 incubator. The time lapse in seconds is shown on the bottom right and the scale bar is 10 μm.

**Video 2**

**Figure 1** –**4**. Whole-ear immunofluorescence scans. The nuclei stained with Hoechst are shown in blue and CD8 staining is shown in green. The figures refer to Fig. 4C and are high resolution versions. Scale bars are 1 mm.
