# Supplementary figures and images for: Midline 1 controls polarization and migration of murine cytotoxic T cells
Source: Immun Inflamm Dis. 2015 Jan 27;2(4):262–71. doi: 10.1002/iid3.44 (PMC4386920; doi:10.1002/iid3.44)

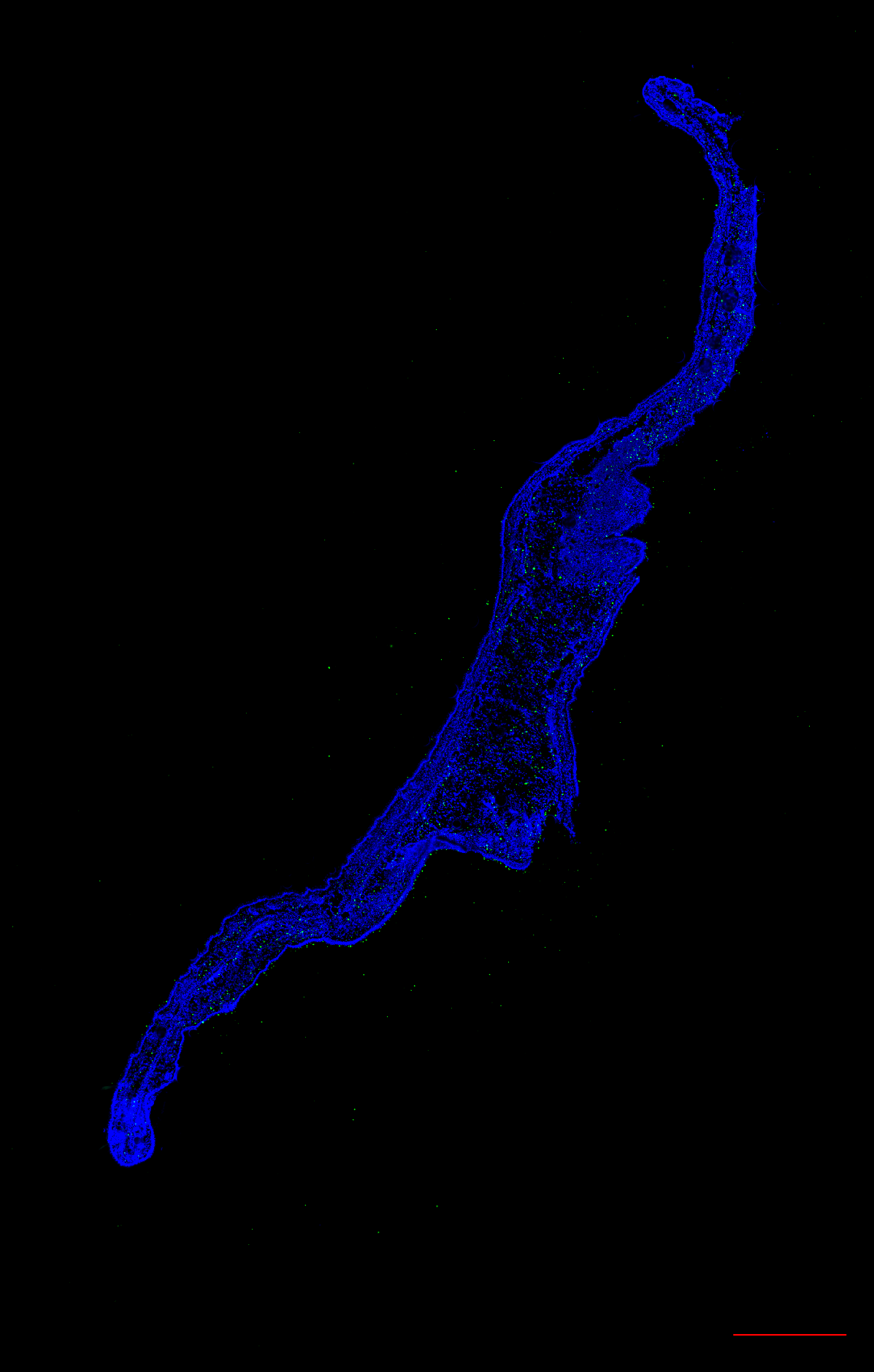

Supplement: Supplementary file 3 [file iid30002-0262-sd3.tif]

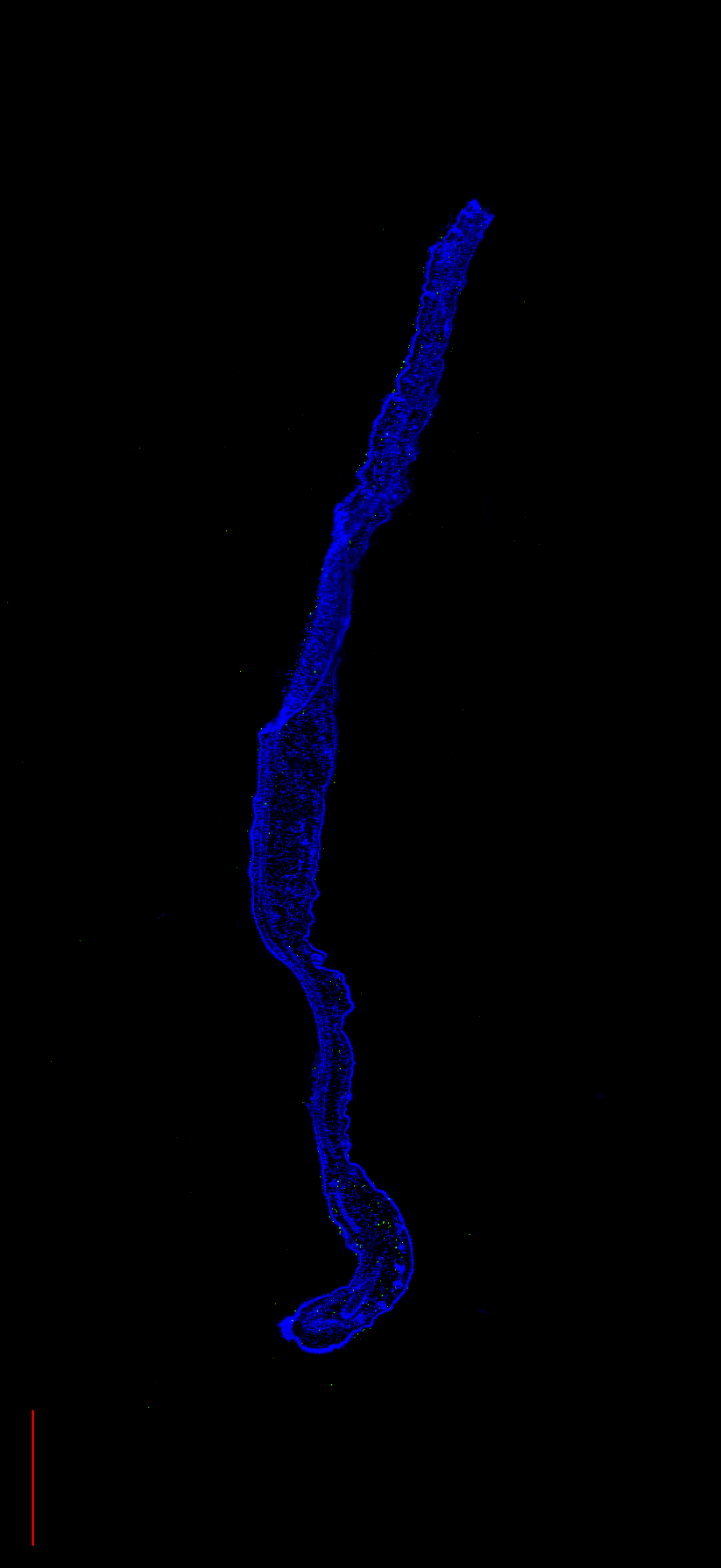

Supplement: Supplementary file 4 [file iid30002-0262-sd4.tif]

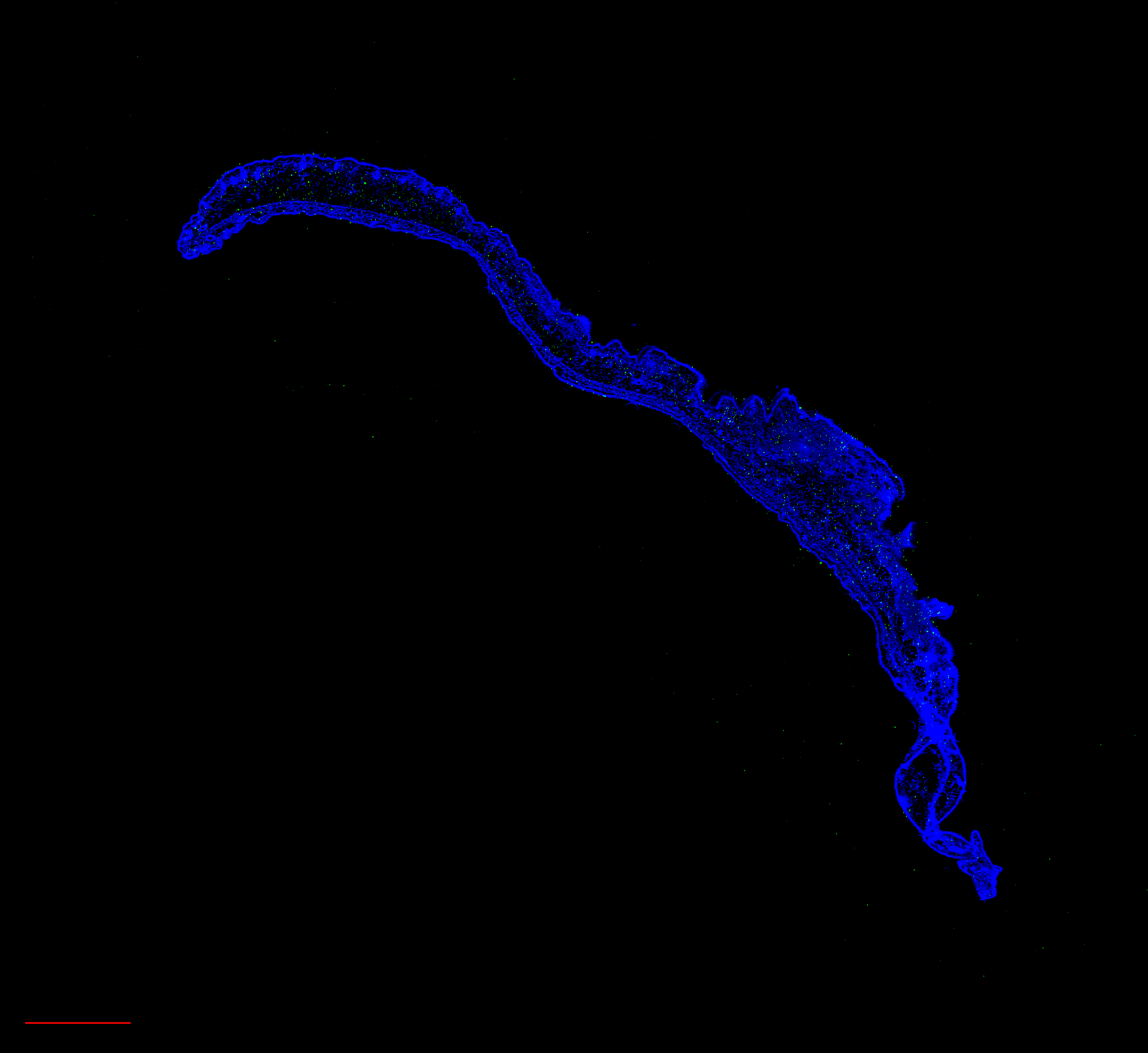

Supplement: Supplementary file 5 [file iid30002-0262-sd5.tif]

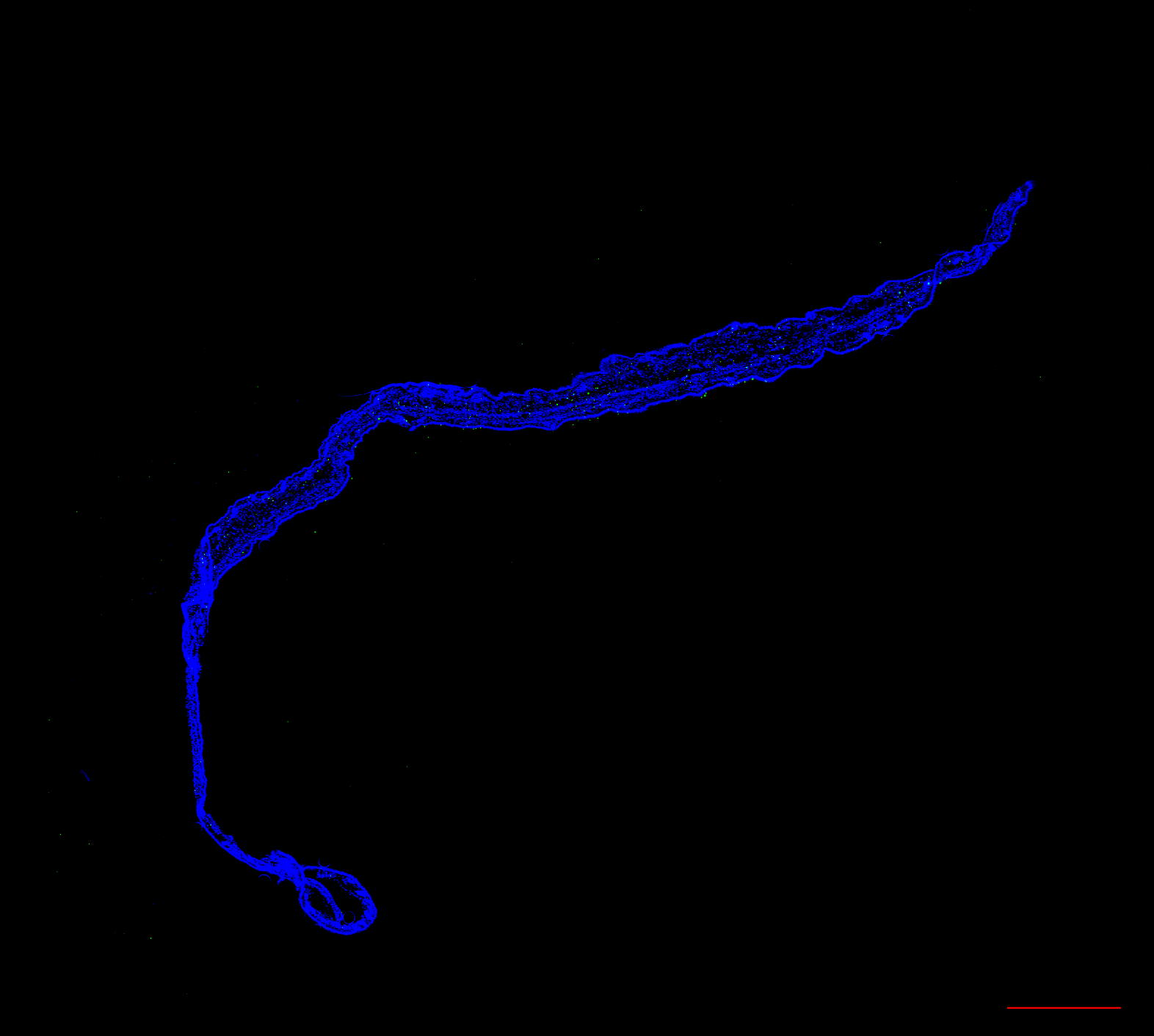

Supplement: Supplementary file 6 [file iid30002-0262-sd6.tif]
